# Human Umbilical Cord Mesenchymal Stem Cells and Derived Hepatocyte-Like Cells Exhibit Similar Therapeutic Effects on an Acute Liver Failure Mouse Model

**DOI:** 10.1371/journal.pone.0104392

**Published:** 2014-08-07

**Authors:** Ruiping Zhou, Zhuokun Li, Chengyi He, Ronglin Li, Hongbin Xia, Chunyang Li, Jia Xiao, Zhi-Ying Chen

**Affiliations:** 1 Laboratory for Gene and Cell Therapy, Shenzhen Institute of Advanced Technology, Chinese Academy of Sciences, Shenzhen, China; 2 Department of Immunobiology, Institute of Tissue Transplantation and Immunology, Jinan University, Guangzhou, China; 3 Guanghua School of Stomatology, Sun Yat-sen University, Guangzhou, China; 4 Department of Stomatology, The 5^th^ Affiliated Hospital, Sun Yat-sen University, Zhuhai, China; 5 Department of Anatomy, The University of Hong Kong, Hong Kong, China; 6 Department of Stomatology, Shenzhen Seventh (Yantian District) People’s Hospital, Shenzhen, China; Cincinnati Children’s Hospital Medical Center, United States of America

## Abstract

Mesenchymal stem cells (MSCs) have exhibited therapeutic effects in multiple animal models so that are promising liver substitute for transplantation treatment of end-stage liver diseases. However, it has been shown that over-manipulation of these cells increased their tumorigenic potential, and that reducing the *in vitro* culture time could minimize the risk. In this study, we used a D-galactosamine plus lipopolysaccharide (Gal/LPS)-induced acute liver failure mouse model, which caused death of about 50% of the mice with necrosis of more than 50% hepatocytes, to compare the therapeutic effects of human umbilical cord MSCs (hUCMSCs) before and after induction of differentiation into hepatocyte (i-Heps). Induction of hUCMSCs to become i-Heps was achieved by treatment of the cells with a group of growth factors within 4 weeks. The resulted i-Heps exhibited a panel of human hepatocyte biomarkers including cytokeratin (hCK-18), α-fetoprotein (hAFP), albumin (hALB), and hepatocyte-specific functions glycogen storage and urea metabolism. We demonstrated that transplantation of both cell types through tail vein injection rescued almost all of the Gal/LPS-intoxicated mice. Although both cell types exhibited similar ability in homing at the mouse livers, the populations of the hUCMSCs-derived cells, as judged by expressing hAFP, hCK-18 and human hepatocyte growth factor (hHGF), were small. These observations let us to conclude that the hUCMSCs was as effective as the i-Heps in treatment of the mouse acute liver failure, and that the therapeutic effects of hUCMSCs were mediated largely via stimulation of host hepatocyte regeneration, and that delivery of the cells through intravenous injection was effective.

## Introduction

Acute liver failure is a catastrophic insult to the liver within a short period of time. It is a life-threatening condition frequently ending up with the patients’ death of multi-system failure such as coagulopathy and encephalopathy [1]. Viral infection (e.g. hepatitis B virus, HBV), drug intoxication (e.g. acetaminophen and halothane), autoimmune hepatitis, sepsis, and Wilson’s disease are common causes of acute liver failure. In the U.S., the most common cause is acetaminophen toxicity, followed by other drug-induced injuries [2]. Currently, liver transplantation is the only effective therapy [3]. However, global shortage of donor liver and rejection of the transplant significantly limit its application.

Transplantation of mesenchymal stem cells (MSCs) from different organ sources has been shown to ameliorate acute liver failure, raising the hopes that MSCs can be used as a liver substitute for treating acute liver failure. Human umbilical cord MSCs (hUCMSCs) are proven to be capable of differentiation into hepatocyte-like cells (i-Heps) with typical hepatocyte functions, e.g. secretion of albumin and storage of glycogen [4]. It has also been shown that hUCMSCs could secret multiple cellular factors to stimulate host hepatocyte proliferation via a paracrine mechanism, promoting the recovery of host liver [5]–[7]. However, one of the most important concerns in application of stem cells is their carcinogenic potential, particularly those that have undergone long term *in vitro* manipulation. It was shown, for example, that spontaneous malignant transformation occurred in about half of the bone marrow-derived human MSCs that had undergone long term culture [8]. Moreover, several studies pointed out that the MSCs played some roles in promoting host cell malignant transformation [9], [10], cancer initiation and metastasis [11], [12]. However, there were also studies suggesting that MSCs were able to suppress the malignant phenotypes of multiple human liver cancer cell lines [13] and leukemia cell lines [14]. Based on these conflict results of MSCs, we hypothesized that reduction of *in vitro* manipulation of these cells before transplantation should significantly reduce their carcinogenetic risk. Although a large number of studies have demonstrated the disease amelioration effects of either hUCMSC or i-Hep, few studies have compared side-by-side the therapeutic effects of these two cell types. In the present study, we used an acute liver failure mouse model to compare side-by-side the liver repair activity of hUCMSCs and i-Heps and study the underlying mechanisms, such as if the long term *in vitro* induction of differentiation to i-Heps was necessary and if the MSCs or i-Heps delivery via tail vein injection effective.

## Materials and Methods

### Isolation and expansion of hUCMSCs

All clinical procedures followed the protocols approved by the ethical committee of Shenzhen Institute of Advanced Technology, Chinese Academy of Sciences. All participants provided their written consents for the current study. Umbilical cords were obtained from Shenzhen Nanshan Hospital (Guangdong, China) from women delivering full-term infants (n = 10). Shortly after baby-delivery, the cords were collected and stored in 0.9% NaCl solution. Upon the removal of the umbilical vein, arteries, and mucous membrane tissues, mesenchymal tissues were cut into 2–3 mm pieces and centrifuged at 300 x*g* for 50 minutes at room temperature. Isolated tissues were cultured in Hank’s balanced salt solution containing 1 mg/ml collagenase type I and penicillin-streptomycin solution (Gibco, Carlsbad, CA) for 4–5 days in regulator cell CO_2_ incubator. For further expansion, the cells were trypsinized, washed with Dulbecco’s phosphate-buffered saline (Gibco), and pelleted by centrifugation at 840 x*g* for 5 minutes. Then, the isolated cell pellets were cultured in 10 cm^2^ culture dishes (Corning Incorporation, Corning, NY) with expansion medium (DMEM/F-12) supplemented with L-glutamine (Gibco), penicillin-streptomycin and fetal bovine serum at a final concentration of 10% (Gibco). Half of the expansion medium was replaced with fresh medium every 3 days. At 90% of confluence, usually 9 days after seeding, cells were trypsinized, diluted and cultured continuously in the expansion medium.

### Induction of i-Hep differentiation

To induce the hepatogenic differentiation, hUCMSCs from passages 2 to 3 were seeded at a density of 1.5×10^4^ cells/cm^2^ of 6- or 24-well plates. In the first 2 weeks, hUCMSCs were cultured in DMEM/F-12 medium with 1 µg/ml hepatocyte growth factor (PeproTech, Rocky Hill, NJ), 10 µg/ml epidermal growth factor (R&D systems, Minneapolis, MN), 1× insulin transferrin selenium solution (Gibco) and 5 mg/ml dexamethasone sodium phosphate (Fluka, Buchs SG, Switzerland). In the following 2 weeks, cells were cultured in the same medium supplemented with 100 µg/ml oncostatin M (Sigma-Aldrich, St. Louis, MO). Culture medium was refreshed every 3–4 days during the 4-week’s differentiation period.

### Induction of adipogenic and osteogenic differentiation

To induce the adipogenic differentiation, hUCMSCs from passages 2 to 3 were seeded at a density of 1.5×10^4^ cells/cm^2^ of 6- or 24-well plates with expansion medium supplemented with 1 µM DEX, 10 mg/L insulin and 0.5 mM IBMX (all from Sigma) for 4 weeks. Culture medium was refreshed every 3 days. Lipid vesicles were visualized by Oil Red O staining.

To induce osteogenic phenotype, hUCMSCs from passages 2 to 3 were seeded at a density of 3×10^4^ cells/cm^2^ of 6- or 24-well plates with expansion medium supplemented with 0.1 µM dexamethasone, 10 mM β-glycerophosphate and 50 µg/mL vitamin C (all from Sigma) for 4 weeks. Culture medium was refreshed every 3 days. Calcium deposition was illustrated by Alizarin Red staining.

### FACS determination of hUCMSCs phenotype

The expanded hUCMSCs at passage 2 were trypsinized and resuspended at a concentration of 1×10^6^ cells/ml in PBS supplemented with 0.5% bovine serum albumin (BSA). Subsequently, the cells were incubated at 4°C for 20 minutes after addition of individual antibodies against different biomarkers, including the mesenchymal stem cell markers CD90-FITC (fluorescein isothiocyanate) and CD-73PE (phycoerythrin) and CD105-APC (allophycocyanin), the extracellular matrix element markers CD44-FITC and CD29-PE, the hematopoietic cell markers CD45-FITC and CD34-PE and CD19-APC and CD14-FITC, and the major histocompatibility complex II marker HLA-DR-PerCP. All antibodies were purchased from BD Biosciences (San Jose, CA). To terminate the reaction, the cells were washed once and resuspended in 300 µl Cell Fix (BD Biosciences). Negative controls included unstained hUCMSC and hUCMSC incubated with respective isotypes coupled with FITC, PE, APC, PerCP, and PE-Cy7 (BD Biosciences). Both stained and unstained cells were analyzed on a FACSCanto II flow cytometer (BD Biosciences) and analyzed with FACSDiva version 6.1.1 software (BD Biosciences).

### Immunofluorescent characterization of i-Hep cells

To evaluate the hepatic functions of differentiated cells, i-Heps cultured on 12-mm round glass cover slips were fixed with 4% formaldehyde (v/v) at room temperature for 15 min and then permeabilized with 1% Triton X-100 in Tris buffer (Gibco) for another 15 min. To block non-specific staining, the cells were treated with PBS buffer containing 5% BSA for 1-hour at 37°C. Subsequently, the cells were incubated in the same solution for 2-hour at room temperature with primary antibodies against human cytokeratin-18 (hCK-18; 1∶100, Abcam HK, NT, HK), human cytokeratin-19 (hCK-19; 1∶100, Abcam HK), human albumin (hALB; 1∶100. Abcam HK) or human α-fetoprotein (hAFP) (1:100, Abcam HK). After three washes with PBS buffer, the cells were incubated for 1-hour with goat antibody against rabbit IgG conjugated with FITC (1:1000, Abcam HK) at room temperature. To illustrate the nuclei, the cells were counter-stained with Hoechst 33342 (Beyotime Institute of Biotechnology, Shanghai, China) for 15-minute at room temperature. The slides were mounted with fluorescent mounting medium (KPL, Gaithersburg, MD) before examination under inverted fluorescent microscope IX71 (Olympus microscope, Tokyo, Japan).

### Periodic acid–Schiff (PAS) staining of i-Hep cells

To evaluate the glycogen storing ability of the cells, i-Heps cultured with or without supplementation of 5 mg/ml α-amylase (Sigma) were fixed with 4% formaldehyde, incubated with 1% periodic acid (Sigma) for 10-minute, followed 3 washes with PBS. To illustrate the nuclei, the slides were incubated with Schiff’s reagent (Sigma) for 15-minute. The stained cells were examined under light microscope (Leica Microsystems Ltd., Milton Keynes, UK).

### Urea production assay

Urea is produced by hepatocyte as the major end product of nitrogen metabolism and urea production assay is frequently used as an indicator of hepatocyte function. hUCMSCs and i-Heps were cultured with William’s medium E (Gibco) for 24-hour supplemented with or without 0.3 mM NH_4_Cl (Sigma). Urea concentration in the media was quantified using urea colorimetric assay kit (BioVision, San Francisco, CA) according to the manufacturer’s instructions. Serum-free Williams’ medium E served as the negative control.

### Animal experiment

All animal experiments, including procedures, sampling and animal cares, in the current study were approved by the ethical committee of Shenzhen Institute of Advanced Technology, Chinese Academy of Sciences. Male 6-week old (∼20 g) non-obese diabetic severe combined immune-deficient (NOD/SCID) mice were bought from Guangdong Experimental Animal Center (Guangzhou, China). We used NOD/SCID instead of SCID mice because the they have lower natural killer (NK) cell activity so that is a better model for study involving human cell transplantation [15]. Mice were randomly divided into 6 groups (n = 7): (1) control group: mice were intraperitoneally (i.p.) injected with PBS only; (2) Gal/LPS group: mice were i.p. injected with 600 mg/kg D-galactosamine (Gal, Sigma) and 8 µg/kg lipopolysaccharide (LPS, Sigma) dissolved in PBS simultaneously; (3) vehicle-hUCMSC group: mice were injected through tail-vein (t.v.) with 2×10^6^ hUCMSCs at passage 2; (4) vehicle-i-Hep group: mice were t.v. injected with 2×10^6^ i-Heps; (5) Gal/LPS-hUCMSCs group: mice were given with 600 mg/kg Gal and 8 µg/kg LPS via i.p. injection, followed 6-hour with 2×10^6^ hUCMSCs at passage 2 through t.v. injection; (6) Gal/LPS-i-Hep group: mice received 600 mg/kg Gal and 8 µg/kg LPS via i.p. injection, followed 6-hour later by 2×10^6^ i-Heps through t.v. injection. The dosage combination of Gal and LPS was selected based on a pilot study that induced approximately half of the mice death (sub-lethal dose) (data not shown). Selection of tail vein injection route, more convenient and clinic relevant because it equals to routine peripheral vein injection, was based on our pilot study demonstrating that hUCMSCs delivered through tail vein, portal vein, or splenic injection exhibited similar therapeutic effects of the intoxicated mice (data not shown). Furthermore, several earlier reports also found that MSC delivered through tail vein injection significantly improved hepatic functions of failing livers [16], [17]. Mouse serum was collected at day 1, 3, 6 and 14 post-cell transplantation. Liver samples were collected at the end of the 14-day experiment and stored at −80°C until further processing.

### Immnuological and histological assays of the liver tissue

Liver tissue samples were fixed in 10% phosphate-buffered formalin, embedded in paraffin, and processed for immunological and histological assays. Five-micrometer tissue sections were cut and stained with hematoxylin and eosin (H&E) or antibodies of hCK-18 and hAFP. For the evaluation of key proteins in host hepatocytes and transplanted stem cells, fresh liver tissues were embedded with optimal cutting temperature (OCT) medium and “snap-frozen” in dry ice. Frozen sections of 10-µm thickness were prepared and subjected to permeabilization in acetone at −20°C for 10 min. To reduce non-specific signal, slides were incubated with goat serum blocking buffer (Boster, Wuhan, China) at room temperature for 1-hour. Subsequently, the slides were incubated with primary antibodies PCNA (1∶100, Cell Signaling Technology, Danvers, MA) and hHGF (1∶500, Takara, Shiga, Japan), respectively. After washing thrice with PBS, slides were incubated with goat antibody against rabbit IgG conjugated with FITC (1:1000, Abcam HK) or mouse antibody against mouse IgG conjugated with Alexa flour (1:1000, Cell Signaling) at room temperature for 1-hour. Hoechst was applied to counter-stain the nuclei at room temperature for 15-minute before examination.

### Serum biochemical measurements

Serologic assays for determining activities of alanine aminotransferase (ALT), aspartate aminotransferase (AST), lactate dehydrogenase (LDH) and level of bilirubin were conducted using serums freshly collected from the mice at termination of the experiments. The following kits were used for individual measurements: ALT (SGPT) reagent set (Teco diagnostics, Anaheim, CA), AST (SGOT) reagent set (Teco diagnostics), LDH kinetic kit (Teco diagnostics) and Direct Bilirubin kit (Teco diagnostics).

### Genomic DNA extraction and quantitative real-time PCR

To quantify the transplanted hUCMSCs and i-Heps that homed at the mice liver, a recently established real-time PCR quantification system has been used in the current study [18]. Briefly, genomic DNA was extracted from mouse livers using QIAamp genomic DNA extraction kit (Qiagen, Hilden, Germany). A pair of primers (forward: 5′-ATGCTGATGTCTGGGTAGGGTG-3′, reverse: 5′-TGAGTCAGGAGCCAGCGTATG-3′) that generate a 141-bp fragment of human Down syndrome region at chromosome 21 were used to quantify the human-derived cells. The real-time PCR reaction was performed using an ABI 7500 real-time PCR system (Applied Biosystems, Foster City, CA) for 40 cycles with denaturing at 95°C for 30 seconds and annealing at 63°C for 34 seconds, with a SYBR-Green Realtime PCR mix (Takara, Dalian, China).

### Statistical analysis

Data from each group were expressed as means ± SEM. Statistical comparison between groups was done using the Kruskal–Wallis test followed by Dunn’s post hoc test to detect differences in all groups. A value of p<0.05 was considered to be statistically significant (Prism 5.0, Graphpad software, Inc., San Diego, CA).

## Results

### Characterization of hUCMSCs

**Figure 1:**
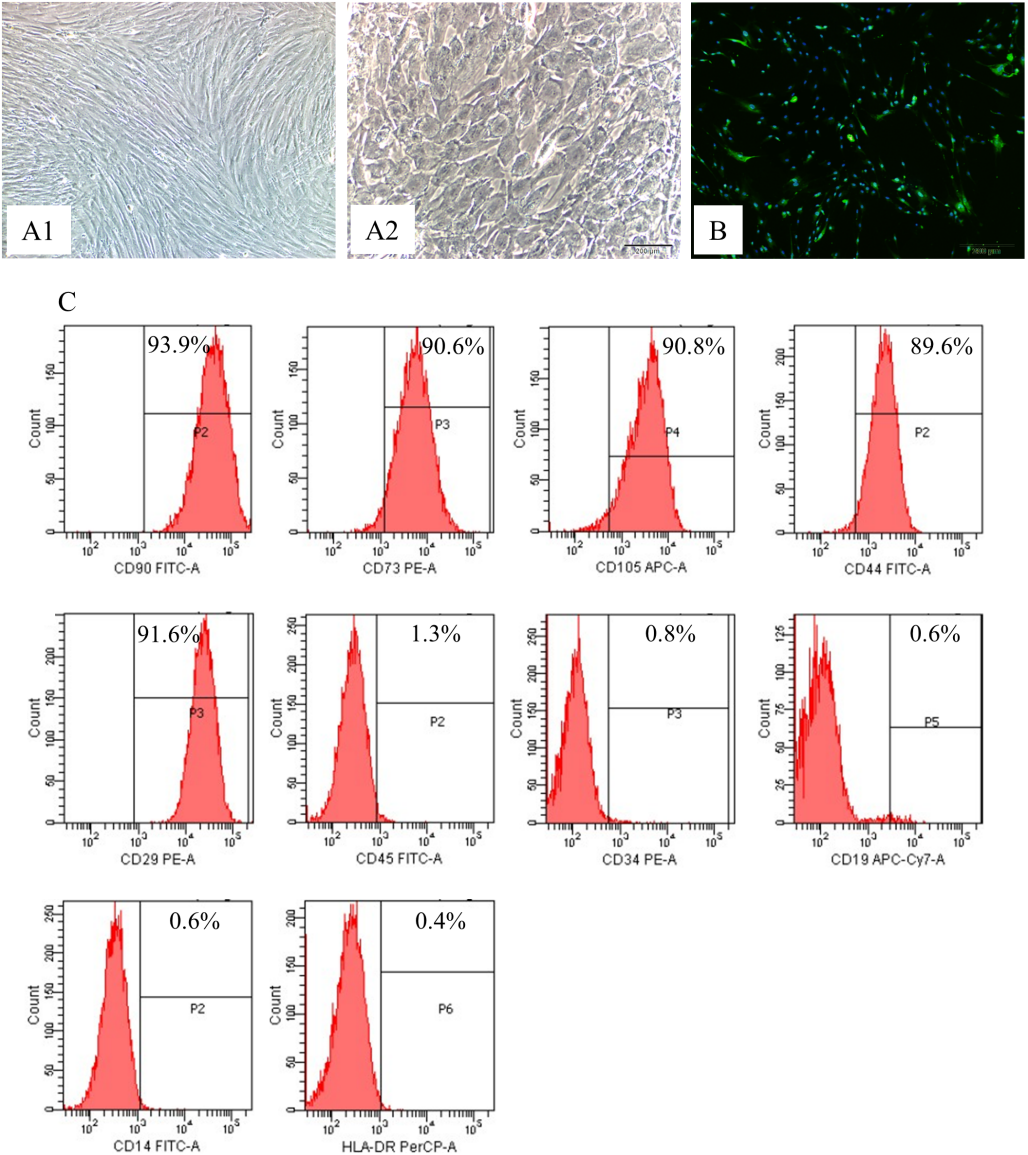
Characterization of hUCMSCs and i-Hep. (A1) hUCMSCs, bright field, x200; (A2) iHep, bright field, x200. (B) Demonstration of mesodermal marker vimentin in hUCMSCs, x200. (C) Flow cytometry analysis of hUCMSCs at passage 2. The hUCMSCs at passage 2 were subjected to immunocytostaining with individual antibodies before subjected to FASC analysis. More than 90% of the cells were positively stained with stem cell markers CD90-FITC-A, CD73-PEA and CD105-PECA; and extracellular matrix makers CD44-FITCA and CD29-FITCA; but more than 98% of the cells are negative in staining with hematopoietic stem cell markers CD45-FITCA, CD34-PEA, CD19-APC-CY7-A and CD14-FITC-A; almost all the cells were negative in staining of the major histocompatibility complex HLA-PRE-CPA. Results are expressed as percentage of the positive cells (%).

Three days after isolation from Wharton’s jelly, hUCMSCs showed a fibroblast-like appearance (Fig. 1A1), which was totally different from the morphology of hepatocyte-like hUCMSCs after 4-week’s induction of hepatogenic differentiation (Fig. 1A2). Immunocytochemical detection of vimentin confirmed the mesodermal origin of hUCMSCs (Fig. 1B).

FACS examination demonstrated that the hUCMSCs expressed high levels of typical MSCs markers, including CD90, CD73 and CD105 as well as extracellular matrix markers CD44 and CD29. In contrast, the levels of hematopoietic cell markers, including CD45, CD34, CD19 and CD14, were very low in these cells. The absence of the major histocompatibility complex II marker (HLA-DR) was also consistent with the typical feature of hUCMSCs [4] (Fig. 1C).

**Figure 2:**
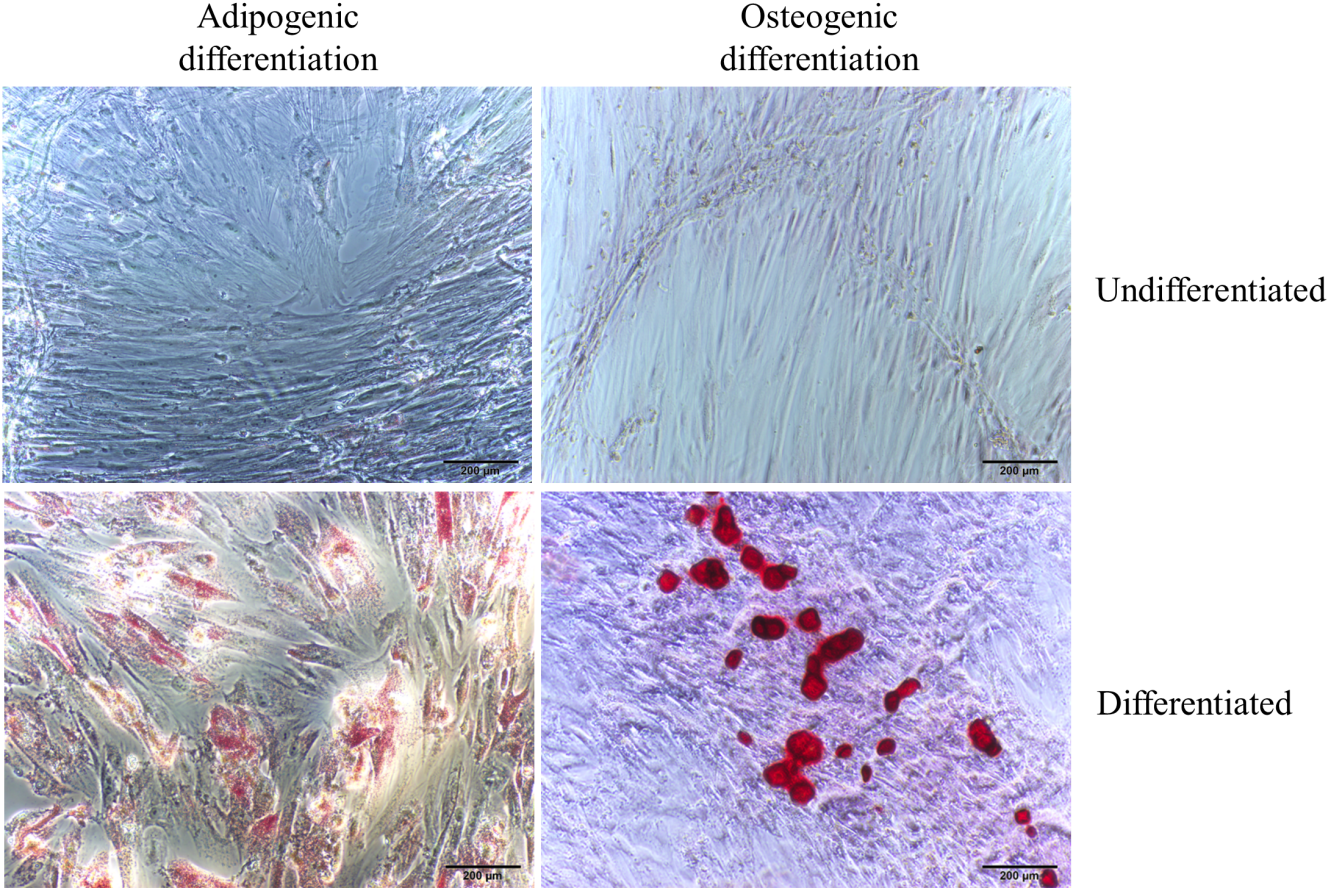
Adipogenic and osteogenic differentiation of hUCMSCs. After 14-day’s induction, adipogenic character of the cells was characterized by formation of intracytoplasmic lipid droplets which became red in color and easily to be identified when stained with Oil Red O. Osteogenic character of hUCMSCs was shown by formation of multiple red bone nodules outside the cells when stained with Alizarin Red. 200x.

After 14-day induction of adipogenic differentiation, hUCMSCs cells exhibited evident lipid droplet accumulation within the cells as illustrated by Oil Red O staining (Fig. 2). Osteogenic differentiation was also achieved as evidenced by the calcium deposition in cell culture exhibited by Alizarin Red staining (Fig. 2). Taken together, these results confirmed that the hUCMSCs we prepared had stem cell-like characters.

### Demonstration of hepatocyte functions of i-Heps *in vitro*

**Figure 3:**
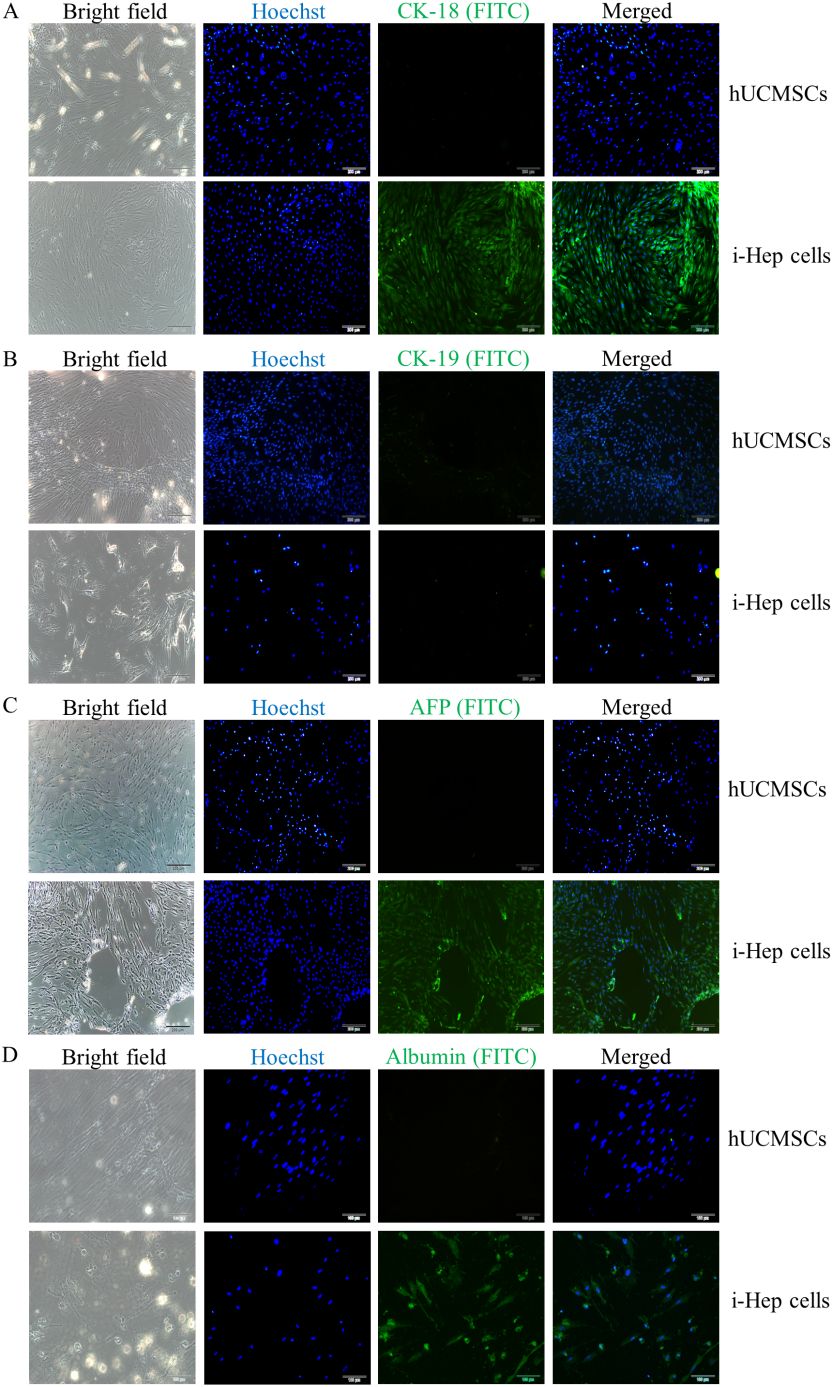
Determination of hepatocyte biomarkers of hUCMSCs and i-Hep *in vitro*. After 14-day’s induction of hepatocyte differentiation, the treated cells were stained with immunofluorescence technique using individual human antibodies. The treated cells were positive while the untreated hUCMSCs negative of the following biomarkers: (A) hCK-18, (B) hCK-19, (C) hAFP, and (D) hALB. hCK-19 was negative in both cell types. These results confirmed that the treated cells had obtained a pattern in expressing a panel of genes which is consistent with the identity of hepatocyte lineage and could be classified as i-Hep. Nuclei were stained with Hoechst 33342. 200x.

**Figure 4:**
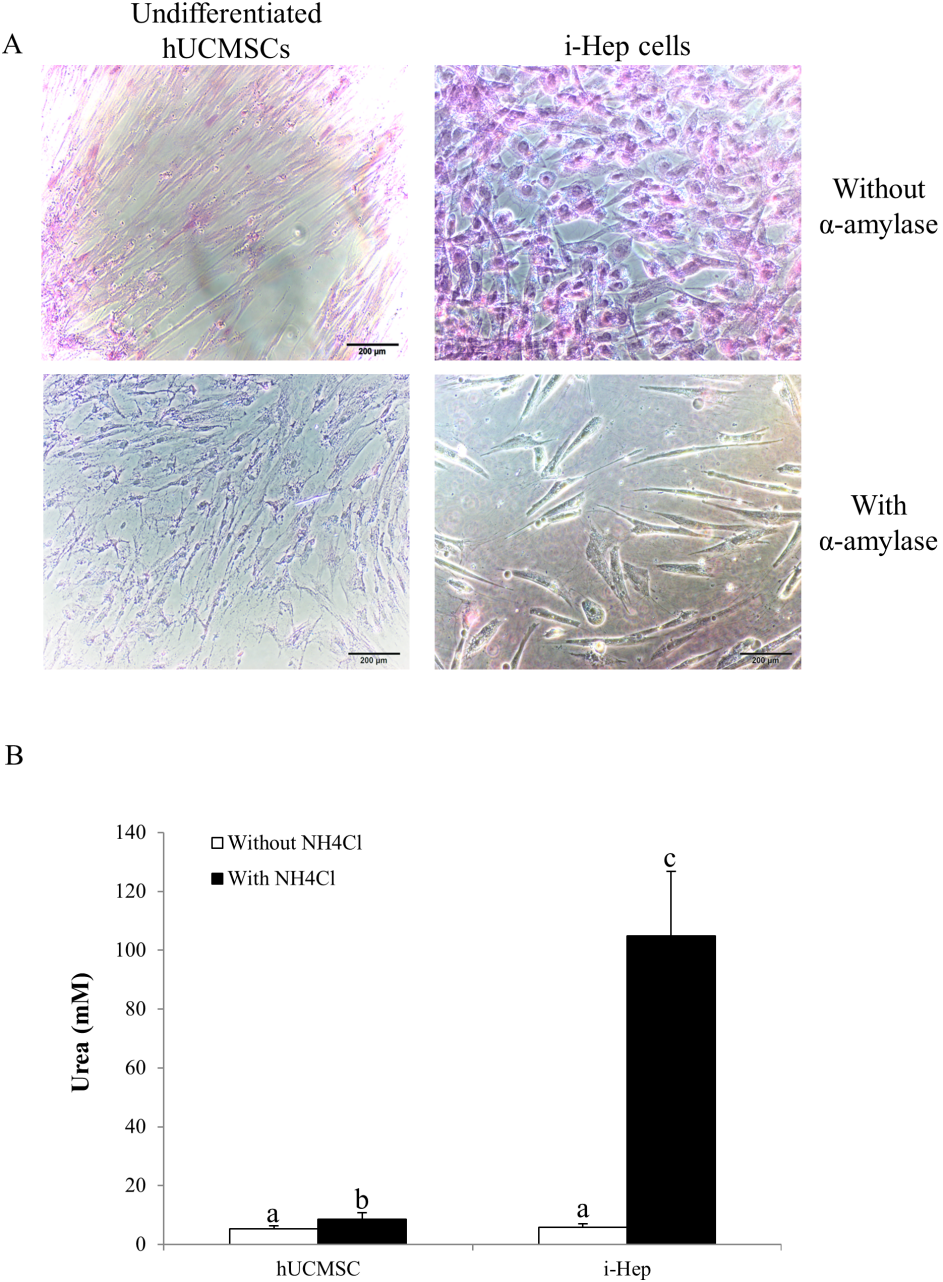
Hepatic metabolism functions of hUCMSCs and i-Hep *in vitro*. (A) In the culture free of α-amylase, hUCMSCs showed modest accumulation of glycogen, while i-Hep demonstrated increased amount of glycogen. In the existence of the α-amylase, no glycogen was seen in either cell types. Periodic acid–Schiff staining, 200x. (B) When supplementation of NH_4_Cl in the medium, i-Hep produced more than 12-fold of urea than hUCMSCs (*p*<0.05).

To induce the differentiation of hUCMSCs into functional hepatocyte-like cells (i-Heps), hUCMSCs were successively exposed to growth factors, cytokines, and chemicals that mimicked developmental liver environment for 4 weeks. The induced i-Heps showed typical hepatocytes morphology similar to human hepatocytes (Fig. 1A2). Immunofluorescent assays demonstrated that the i-Hep expressed typical markers of human hepatocytes, including hCK-18, hAFP and hALB (Fig. 3A, 3C and 3D), which were absent in the untreated hUCMSCs. Both hUCMSCs and i-Heps showed no expression of the epithelial marker hCK-19 (Fig. 3B), which is often used as a cholangiocyte marker and a negative control of the hepatocyte lineage. Although some studies reported that undifferentiated MSC could express hCK-18, we observed only a very low level of hCK-18 in our hUCMSCs. Since CK-18 is an epithelial marker, conceivably, the hUCMSCs we used, at passage 2, did not reach the differentiation stage capable of expressing significant level of hCK-18 [19]. To evaluate the hepatocyte functions of the i-Heps, we performed PAS staining and urea measurement and found that i-Heps were able to store more glycogen than the hUCMSC (Figure 4A), and that i-Heps were able to produce 12-fold more urea as compare to the hUCMSCs (Figure 4B). It is interesting to observe that hUCMSCs could store a low level of glycogen (Figure 4A), although the mechanism is not clear. Similar observation was also reported in undifferentiated chicken embryonic stem cell [20] and human adipose stem cells [21].

### Both hUCMSCs and i-Heps rescued mice with lethal acute hepatic injury

**Figure 5:**
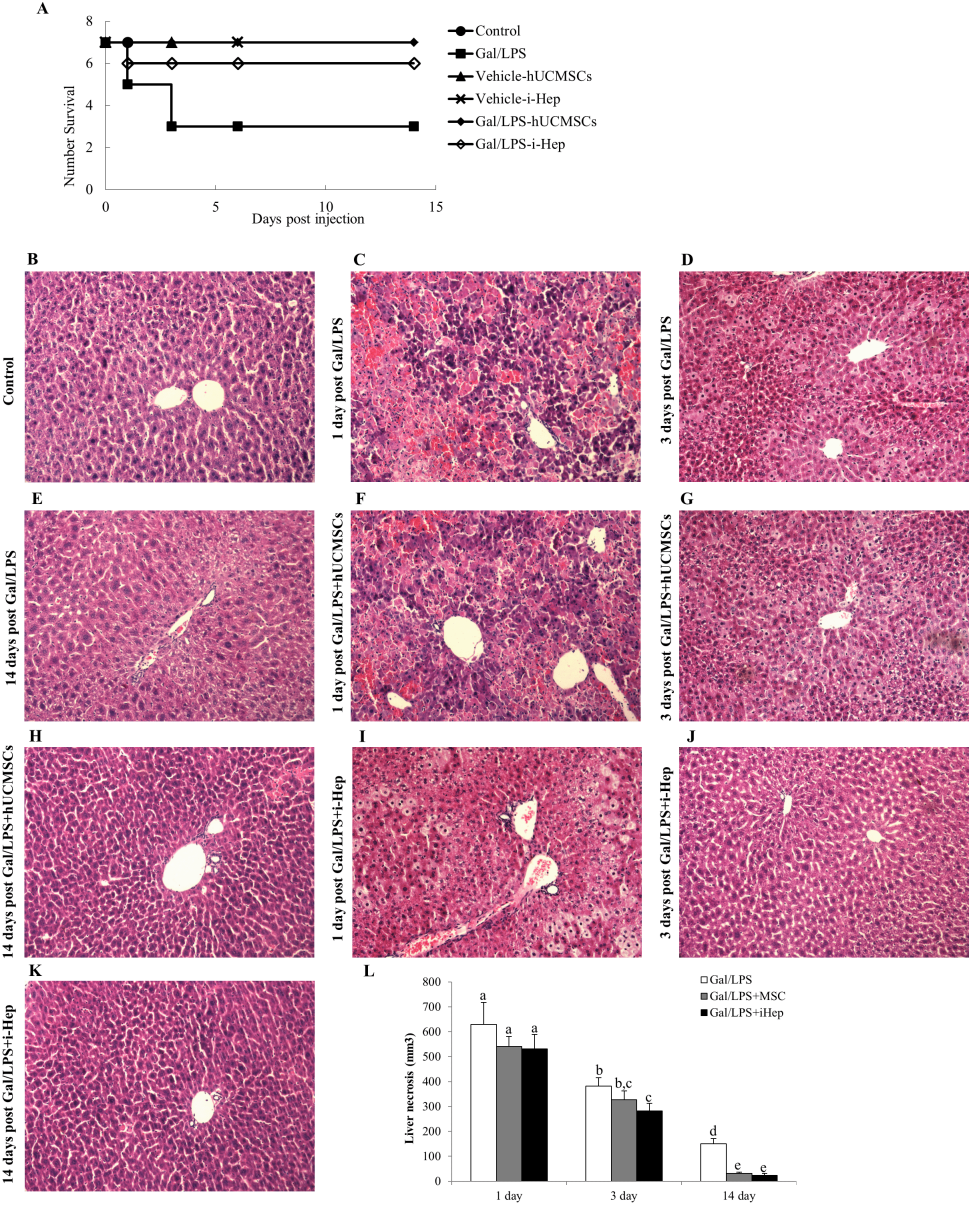
Effect of transplantation of MSCs on mouse liver histology. (A) Survival curve of each group of mice after D-galactosamine/lipopolysaccharide (Gal/LPS) intoxication with or without stem cell administration. Representative images of hematoxylin and eosin (H&E) staining of (B) control, (C, D, E) 1, 3 and 14 days post Gal/LPS treatment, respectively; (F, G, H) 1, 3 and 14 days post Gal/LPS treatment with transplantation of human umbilical cord mesenchymal stem cells (hUCMSCs), respectively; (I, J, K) 1, 3, 14 days post Gal/LPS treatment with induced-differentiation of hepatocytes (i-Hep), respectively; and (L) Double-blind quantification of necrotic area of the hepatic histology of each group. Data were analyzed with ImageJ software and expressed as means ± SEM. Different superscript letters (e.g. a vs. b, or b vs. c) represent a statistically significant difference (*p*<0.05) between two variables. Magnification 200x.

In a pilot study, we found that co-treatment of 600 mg/kg Gal and 8 µg/kg LPS caused death of approximately half of the NOD/SCID mice within 3 days. Therefore, we defined these dose levels as the “sub-lethal dose” of Gal/LPS treatment and used it as the acute liver failure protocol to test the therapeutic efficacy of transplanted MSC. Both hUCMSCs and i-Heps were transplanted 6-hour following the liver intoxication. Fourteen days post-injection, only 3 mice of the Gal/LPS group (n = 7) survived. Transplantation of the hUCMSCs rescued all the intoxicated mice while i-Heps rescued all but one mouse (n = 7). Transplantation of either hUCMSCs or i-Heps in the mice without GAL/LPS pretreatment did not cause any obvious changes of the mice (Figure 5A).

Gal/LPS treatment is known to induce hepatocyte necrosis and inflammatory responses. In the present study, Gal/LPS treatment alone caused significant hepatic injury in the NOD/SCID mice in 24-hour, where the necrosis areas were more than 50% of almost all lobules examined (Figure 5C). Three days after the intoxication, the liver injury remained very evident (Figure 5D). By the end of the experiment (14 days post-injection), however, the mice survived the Gal/LPS-intoxication exhibited a recovered hepatic histology, although some small necrotic and inflammatory cells infiltration sites could be observed (Figure 5E). The therapeutic efficacy of hUCMSCs and i-Hep was also reflected in liver histology. One day after the Gal/LPS-intoxication, there was no significant difference in the severity of liver injury, as judged by necrosis area, among all groups (Figure 5F, 5I and 5L). However, 3 days after the intoxication, hUCMSCs and i-Heps groups showed more significant histological improvement than Gal/LPS-alone group (Figure 5G, 5J and 5L). At 14-day post-intoxication, co-treatment with hUCMSCs or i-Heps exhibited an almost complete normal liver histology (Figure 5H, 5K and 5L). Thus, transplantation with either hUCMSCs or i-Heps not only rescued the mice from death, but also significantly accelerated the recovery as judged by liver histology (Figure 5F and 5G). In the vehicle control groups, injection of either hUCMSCs or i-Heps showed no changes on the liver morphology (data not shown) throughout the experiment.

### Transplantation of either hUCMSCs or i-Heps improved serum biochemistry

To evaluate the therapeutic effects of hUCMSCs transplantation on the Gal/LPS -induced liver injury in serum biochemistry level, four key serum biomarkers, including ALT, AST, LDH and bilirubin levels, were measured at 1-, 3-, 6- and 14-day post-injection of Gal/LPS challenge. One-day and 3-day after Gal/LPS challenge, both ALT and AST levels in the serum of all Gal/LPS-treated mice increased significantly as compared to those of the untreated control mice. However, transplantation of both hUCMSCs and i-Heps significantly alleviated the liver injury indicators ALT and AST (Table 1). The Gal/LPS-treated mice recovered almost completely from the acute liver injury at the end of the 14-day experiment, as evidenced by the full recovery of serum ALT and AST. Similarly, the serum levels of LDH and bilirubin were also significantly increased at different time-points in response to the Gal/LPS treatment (most obvious at day 1 and day 3 post-injection). Transplantation of both kinds of stem cells effectively alleviated these serum injury markers (Table 1).

**Table 1:** Evaluation of serum level of biochemical markers.

Group	Day 0	Day 1	Day 3	Day 6	Day 14
ALT level (IU/L)*					
Control	32.1±2.9^a^**	34.6±2.3^a^	34.9±2.6^a^	30.1±3.8^a^	30.6±3.3^a^
Gal/LPS	30.2±2.6^a^	297.5±33.6^b^	222.8±31.1^b^	85.4±12.7^b^	34.7±7.9^a^
Vehicle-hUCMSCs	29.5±3.2^a^	36.4±3.9^a^	37.0±5.2^a^	27.4±2.1^a^	30.0±2.7^a^
Vehicle-i-Hep	33.0±2.8^a^	31.9±4.1^a^	33.8±3.9^a^	31.9±3.6^a^	29.1±2.8^a^
Gal/LPS- hUCMSCs	31.7±2.2^a^	236.1±31.0^c^	162.8±21.9^c^	41.7±5.5^c^	28.8±2.2^a^
Gal/LPS-i-Hep	32.5±3.4^a^	243.7±28.9^c^	177.3±22.1^c^	50.2±6.3^c^	30.5±3.2^a^
AST level (IU/L)*					
Control	78.8±8.9^a^	70.1±7.0^a^	73.4±8.9^a^	70.8±6.6^a^	75.1±7.8^a^
Gal/LPS	75.5±6.4^a^	695.3±72.2^b^	498.7±57.0^b^	238.5±33.0^b^	81.8±9.5^a^
Vehicle-hUCMSCs	76.7±7.1^a^	66.5±7.8^a^	69.2±8.8^a^	75.7±8.1^a^	74.0±6.0^a^
Vehicle-i-Hep	79.0±8.2^a^	73.5±6.6^a^	67.5±9.2^a^	69.0±6.2^a^	77.9±9.1^a^
Gal/LPS- hUCMSCs	74.0±7.3^a^	486.1±50.0^c^	297.3±32.7^c^	82.2±10.4^a^	70.6±6.7^a^
Gal/LPS-i-Hep	78.5±7.7^a^	511.2±54.9^c^	365.9±36.2^d^	128.6±11.1^c^	78.8±6.5^a^
LDH level (IU/L)*					
Control	134.2±11.1^a^	126.4±14.6^a^	130.7±13.4^a^	111.4±10.8^a^	146.7±18.3^a^
Gal/LPS	127.9±13.2^a^	505.5±54.8^b^	477.1±61.2^b^	259.3±36.9^b^	195.7±34.5^b^
Vehicle-hUCMSCs	129.5±11.7^a^	119.9±11.6^a^	140.2±15.5^a^	127.9±10.2^a^	134.9±12.7^a^
Vehicle-i-Hep	140.5±14.4^a^	122.7±10.1^a^	126.9±12.3^a^	110.1±14.3^a^	116.0±11.2^c^
Gal/LPS- hUCMSCs	135.6±13.0^a^	401.8±44.1^c^	366.3±28.2^c^	138.5±15.5^a^	140.3±13.5^a^
Gal/LPS-i-Hep	128.8±14.2^a^	397.3±38.0^c^	386.0±42.1^c^	116.3±12.2^a^	129.5±15.2^a^
Total bilirubin (mg/dl)*					
Control	0.5±0.1^a^	0.4±0.1^a^	0.5±0.1^a^	0.5±0.1^a^	0.6±0.2^a^
Gal/LPS	0.5±0.1^a^	1.9±0.3^b^	1.5±0.2^b^	0.6±0.2^a^	0.5±0.2^a^
Vehicle-hUCMSCs	0.4±0.1^a^	0.5±0.1^a^	0.5±0.1^a^	0.5±0.1^a^	0.5±0.1^a^
Vehicle-i-Hep	0.5±0.1^a^	0.4±0.1^a^	0.4±0.1^a^	0.4±0.1^a^	0.5±0.1^a^
Gal/LPS- hUCMSCs	0.4±0.1^a^	1.2±0.2^c^	0.6±0.1^a^	0.4±0.1^a^	0.6±0.1^a^
Gal/LPS-i-Hep	0.5±0.1^a^	1.2±0.2^c^	0.8±0.1^c^	0.5±0.1^a^	0.5±0.1^a^

*Values are means ± S.D., n = 3–7.

*Pairs of different superscript letters (e.g. a vs. b, or b vs. c) represent a significant difference between groups at the same day (*p*<0.05 or more significant).

### Engraftment of transplanted stem cells in recipient livers

**Figure 6:**
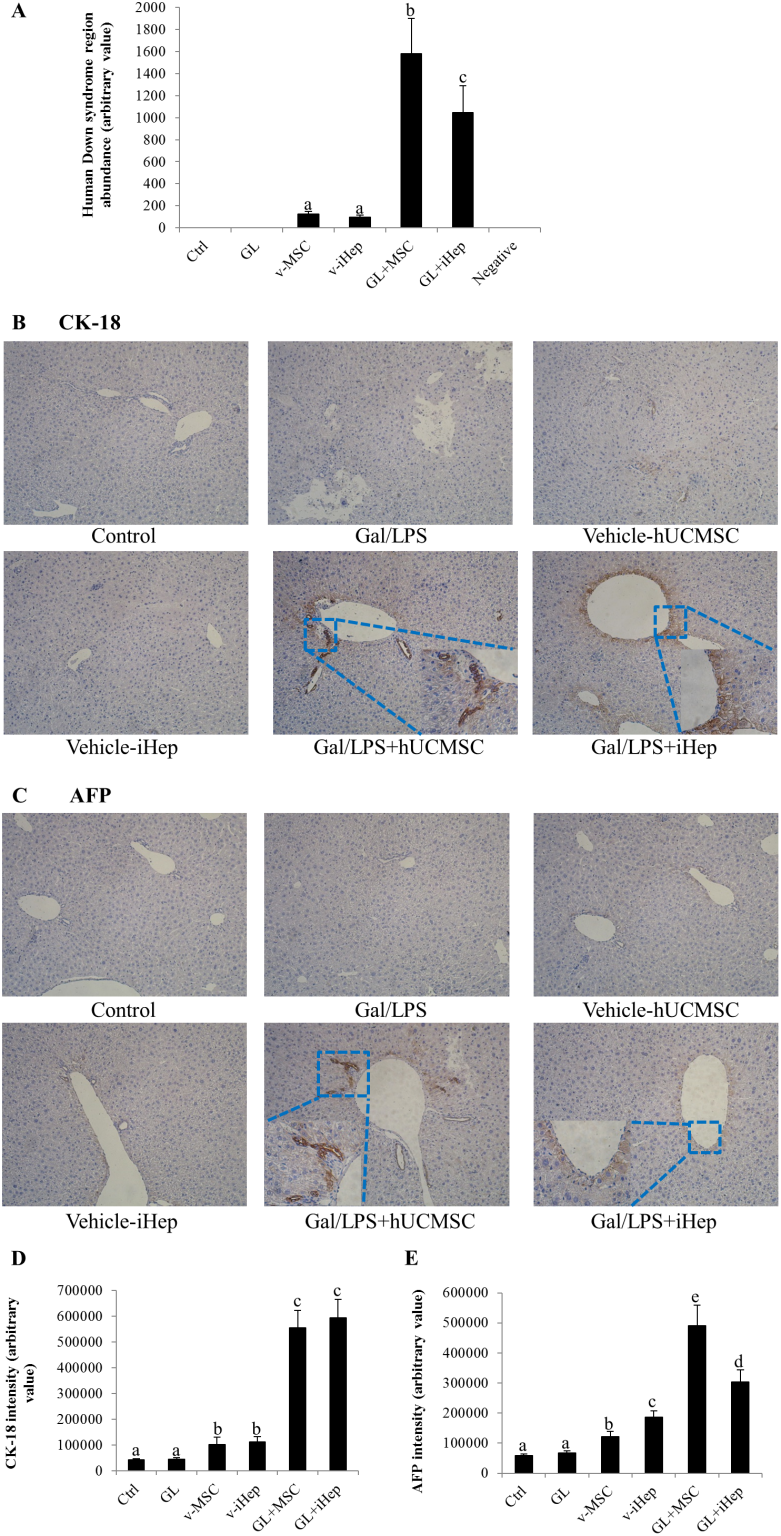
Quantification and function characterization of transplanted stem cells in the mouse livers. (A) Quantitative real-time PCR measurement of engrafted human stem cell in the host liver. The ratios between human genome vs host genome in mouse livers of Gal/LPS- group was 12.6-fold of that of Vehicle-hUCMSCs group (*p*<0.05), and a 10.8-fold difference was demonstrated between Gal/LPS- and Vehicle-i-Hep group (*p*<0.05), respectively. Data were expressed as means ± SEM (n = 3–7). Representative images of (B) hCK-18- and (C) hAFP-positive cells in host livers. Immunohistological staining with individual antibodies against human epitopes. 200x. (D) Expression level of selective human genes in mouse liver was quantified by measuring the intensity of hCK-18- and (E) hAFP-positive cells in liver sections. Data were analyzed with ImageJ software and expressed as means ± SEM. Different superscript letters (e.g. a vs. b, or b vs. c) represent a statistically significant difference (*p*<0.05) between two variables.

To quantify the human-derived cells engrafted in the mouse liver, the ratio between human gene, the Down Syndrome Region Sequence, and host genome in the mouse liver at the end of the experiment was determined using quantitative real-time PCR using liver genome as template. As expected, no human gene was detected in untreated control and Gal/LPS-only groups. Both vehicle-hUCMSC and -i-Hep groups showed a small amount of human gene, transplantation of both human cell types following the Gal/LPS intoxication increased the human-derived cells by 12.6- and 10.8-fold, respectively (Figure 6A). To further investigate *in vivo* differentiation of the engrafted stem cells, cells expressing human hepatocyte markers hCK-18 and hAFP were quantified in immunohistochemical staining liver sections using the ImageJ software. A population of hCK-18- and hAFP-positive cells was demonstrated in both Gal/LSP-hUCMSCs and Gal/LSP-i-Hep groups, occupying less than 10% of the lobules and largely surrounding the central veins (Figure 6B–6E). Because hCK-18 and hAFP are the biomarkers of hepatocyte lineage, these observations suggest that both types of engrafted cells were able to undergo differentiation to become hepatocytes in the injured host liver. However, the accelerated recovery of the injured liver should be attributed largely to the accelerated proliferation of host hepatocytes because the percent of hUCMSCs-derived hepatocytes was small. In contrast, mice transplanted with hUCMSCs or i-Heps without Gal/LPS challenge showed only a very rare hCK-18- and hAFP-positive cells in the liver (Figure 6B–6E), suggesting that an injured liver environment might be in favor of the hUCMSCs to undergo hepatocyte differentiation.

### Transplanted MSCs accelerated host hepatocytes regeneration through secretion of hHGF

**Figure 7:**
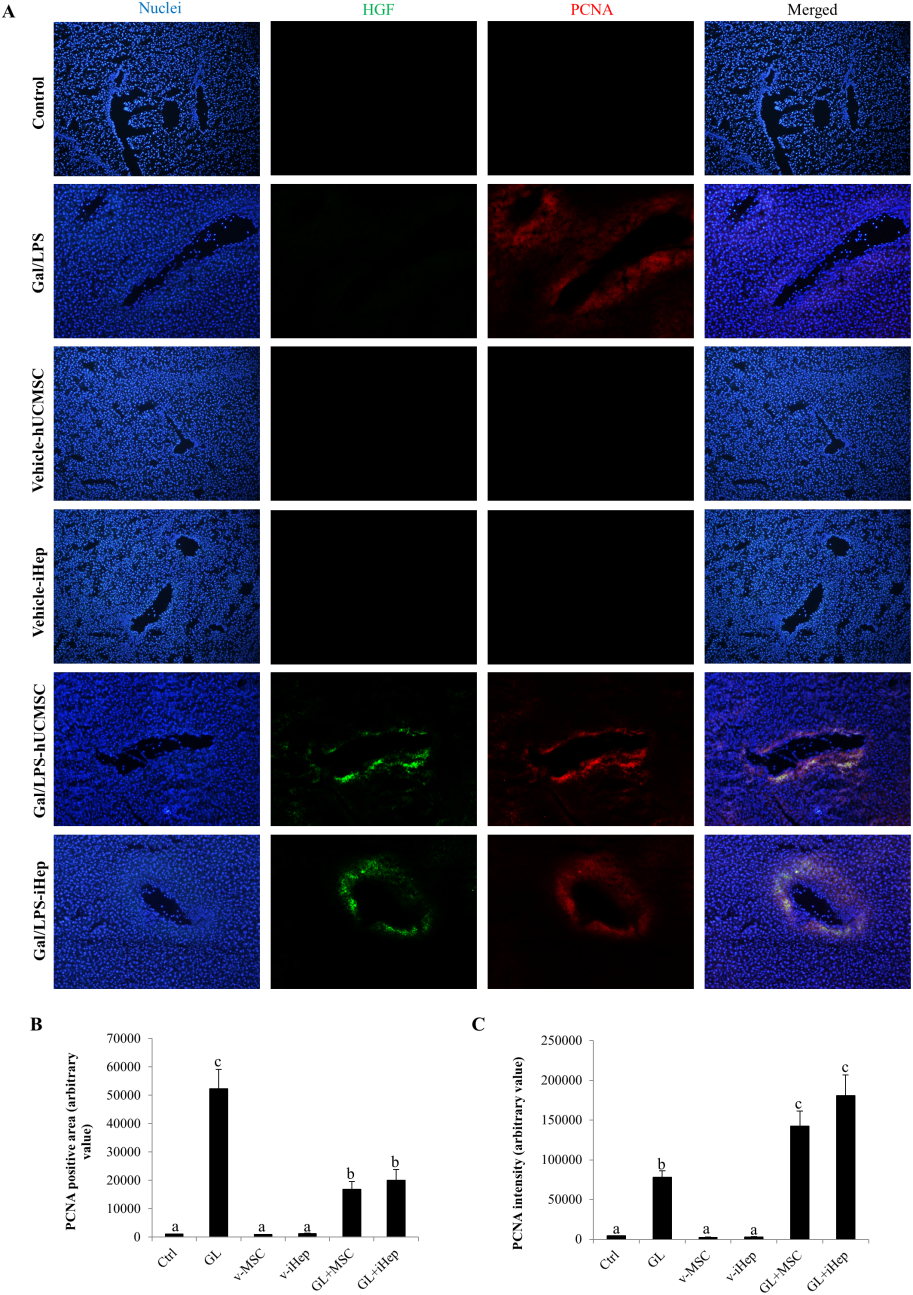
Quantification of expression level of human hepatocyte growth factor (hHGF) of the transplanted stem cells in host liver. (A) Representative images of hHGF-positive cells (green), which was human-specific, and PCNA-positive nuclei (red), which comprised both human and mouse cells. Nuclei were counter-stained with Hoechst 33342 in blue. Magnification 200x. These images were analyzed by ImageJ software, and the area and intensity of PCNA-positive nuclei in each group was exhibited in panel (B) and (C), respectively. Data were expressed as means ± SEM. Different superscript letters (e.g. a vs b, or b vs. c) represent a statistically significant difference (*p*<0.05) between two variables.

To further elucidate the molecular mechanism of hUCMSC- and i-Hep-induced speeding up of liver recovery, double immunofluorescence staining of hHGF and PCNA of mice liver sections was performed. Consistent with the hCK18- and hAFP-expression pattern, there was few hHGF- and PCNA-positive cells in both vehicle-hUCMSCs and vehicle-i-Hep groups. We found that 1 day post-treatment, regeneration reaction, as evidenced by PCNA-positive nuclei, was seen in all Gal/LPS groups, and hUCMSCs or i-Heps administrations did not change the number of the PCNA-positive cells (Figure S1). However, at 3 days post-treatment, both hUCMSCs and i-Hep groups exhibited a weak signal of hHGF in the centrilobular areas (Figure S1). After 14 days, a large PCNA-positive cell population with low intensity could still be observed in the Gal/LPS alone group, indicating that an active liver repairing activity was continuing (Figure 7A–7C). In contrast, in both Gal/LPS-hUCMSCs and Gal/LPS-i-Hep groups, a smaller PCNA-positive cell population with potentiated expression level was seen largely in the centrilobular areas, suggesting that resident hUCMSCs and i-Heps might potentiate host hepatic regeneration. Conceivably, it was the transplanted hUCMSCs and i-Heps that accelerated the recovery process (Figure 7A–7C). Interestingly, the PCNA-positive cell population partially overlapped with the hHGF-positive population, suggesting that hUCMSCs and i-Heps speeding up liver regeneration through the hHGF-mediated paracrine mechanism.

## Discussion

Stem cell-based therapy for end-stage liver diseases received massive attention in recent years because preliminary evidences suggested that it had the potential to overcome the major obstacles of liver transplantation: shortage of donor liver and immune rejection of the transplant. Although progress has been made in this area, many problems have to be solved before the expectation can be met. One of the major problems, for example, is the possible malignant transformation of stem cells. Earlier studies suggested that extensive manipulation of the stem cells might increase the cancer potential while reduction of *in vitro* treatment may minimize the risk. In the present study, we used an acute liver failure model to compare the therapeutic efficacy of hUCMSCs that underwent minimal *in vitro* treatment and the i-Heps that underwent 14-day’s induction and possessed evident hepatocyte functions.

The acute liver failure protocol used in this study comprised a combined treatment of Gal and LPS which is used frequently in experimental hepatology. LPS, also known as lipoglycans or endotoxins, are large molecules produced by Gram-negative bacteria. They are potent immunogens capable of inducing severe immune responses, including liver inflammation and necrosis mediated by the Toll-like receptor 4-dependent immune activation pathway. Gal is a widely used sensitizing agent capable of reducing the median lethal dose (LD_50_) of toxic reagents such as LPS and TNF-α. Its sensitizing effects mainly mediated by blocking RNA and protein synthesis of hepatocyte [22]. In a preliminary experiment, we found that co-treatment with 600 mg/kg Gal and 8 µg/kg LPS caused death of about half of the treated NOD/SCID mice, with more than 50% of hepatic cells underwent necrosis. Therefore, we used this combined Gal/LAS treatment in the present experiment and were able to reproduce the results in both mouse mortality and hepatic injury (Figure 5A and B). Transplantation of either hUCMSCs or i-Heps rescued almost all but one mouse, and significantly accelerating recovery from liver injury as evidenced by hepatic histology and serum biomarkers (Table 1 and Figure 5B). Our observations are consistent with previous studies of Moslem et al [7] and Parekkadan et al [23] who demonstrated that transplantation of MSCs, or their conditioned medium and cell lysate was beneficial to mice with lethal fulminant hepatic failure. Moreover, in a carbon tetrachloride (CCl_4_)-induced mice acute liver model, Burra et al also found that tail vein injection of untreated hUCMSCs accelerated the resolution of acute liver injury [24]. These data strongly suggested that, for the treatment of acute liver failure, hUCMSCs worked as well as i-Heps and long-term *in vitro* manipulation is not needed.

We also studied the mechanisms of the therapeutic effects of both hUCMSC and i-Hep cell types. We demonstrated that hUCMSCs and i-Heps, although delivered through tail vein injection, were able to home at the injured liver as demonstrated by the hCK-18-, hAFP- and hHGF-positive cell populations. Delivery of therapeutic cells through a peripheral intravenous injection will be attractive to clinicians because it is noninvasive and can be conducted as a routine procedure. We also demonstrated scattered hAFP- and hCK-18-positive hepatocytes or progenitors in the centrilobular areas of the recovering mouse livers. Although the size of these cell populations was very small and accounted only for a small fraction of the more than 50% cell loss of the injured livers, these observations do suggest that hUCMSCs have the potential to undergo differentiation into hepatocyte in the *in vivo* environment of the injured livers. Because differentiation usually takes a time longer than the 14-day duration of the current experiment, there may be the possibility that a larger i-Hep population can be produced from hUCMSCs in a chronic liver injury model. We also demonstrated the PCNA-positive populations partially overlapping with the hHGF-positive populations in both Gal/LPS-hUCMSC and -i-Hep groups, suggesting that the increased cell proliferation activity was induced, at least partially, through a paracrine mechanism of hHGF (Figure 7). Our observations agreed with the hypothesis that amplified host residual hepatocytes compensated most of the lost hepatocyte. Previous studies also reported that transplanted MSCs were able to secret a variety of cytokines and growth factors capable of enhancing host hepatocyte proliferation [25]–[27].

In conclusion, in the present study we demonstrated that transplantation of both hUCMSCs and i-Heps, delivered through tail vein injection, exhibited similar therapeutic effects for the mouse acute liver failure. Both cell types were able to home at the injured livers and rescue almost all mice. These observations have important clinical implications in that the hUCMSCs worked as well as the i-Heps in treating the intoxicated mice so that a long-term *in vitro* manipulation is not needed, and that they can be delivered through convenient intravenous injection.

## Supporting Information

Figure S1 **Assessment of production of human hepatocyte growth factor (hHGF) from transplanted stem cells at 1 and 3 days post-injection.** Representative images of human hHGF-positive cells (green) and PCNA-positive nuclei (red) at 1 and 3 days post treatment. Nuclei were counter-stained with Hoechst 33342 in blue. Magnification 200x.
(TIF)
