# Supplementary figures and images for: Human Umbilical Cord Mesenchymal Stem Cells and Derived Hepatocyte-Like Cells Exhibit Similar Therapeutic Effects on an Acute Liver Failure Mouse Model
Source: PLoS One. 2014 Aug 7;9(8):e104392. doi: 10.1371/journal.pone.0104392 (PMC4125182; doi:10.1371/journal.pone.0104392)

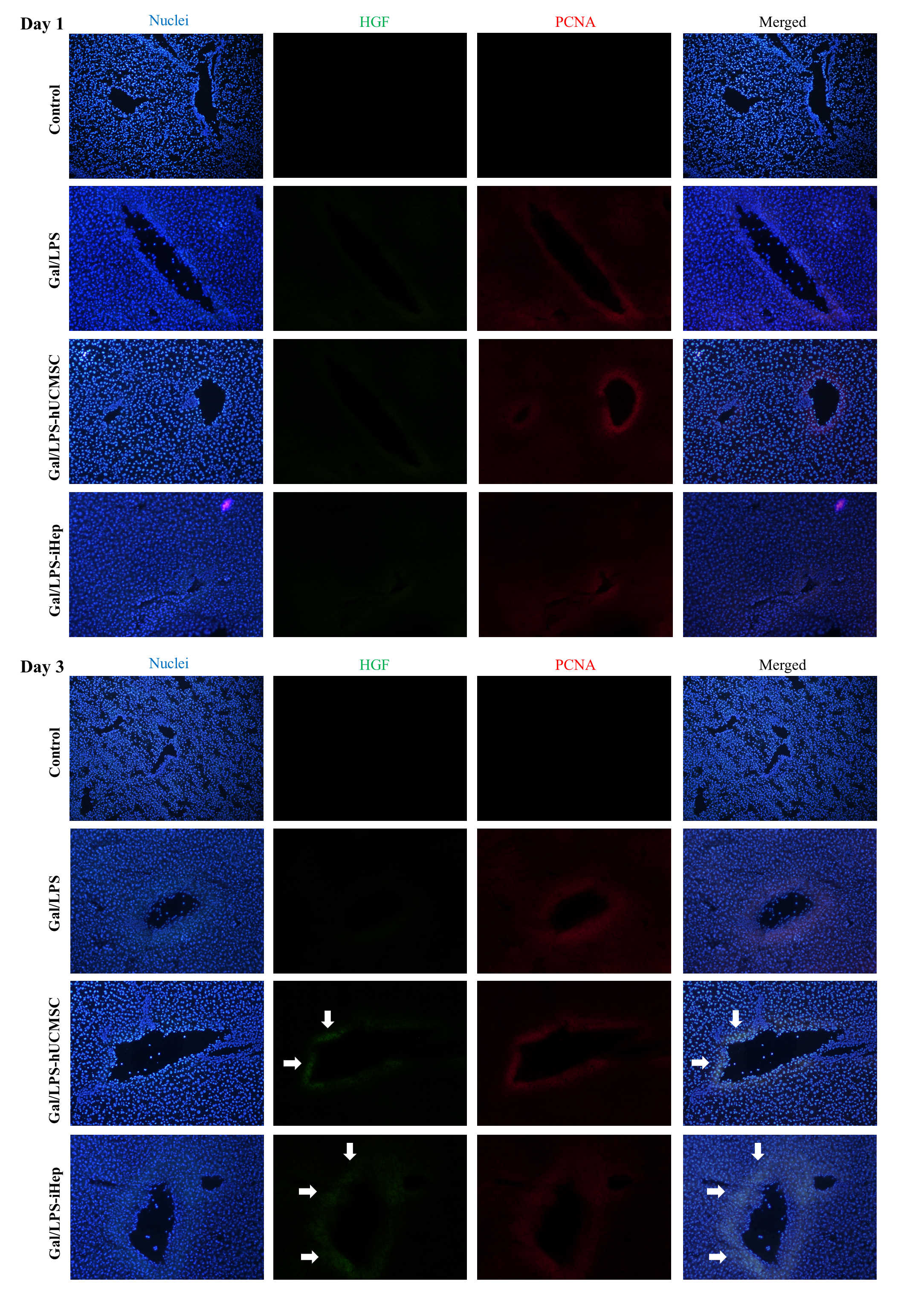

Supplement: Figure S1 — Assessment of production of human hepatocyte growth factor (hHGF) from transplanted stem cells at 1 and 3 days post-injection. Representative images of human hHGF-positive cells (green) and PCNA-positive nuclei (red) at 1 and 3 days post treatment. Nuclei were counter-stained with Hoechst 33342 in blue. Magnification 200x. (TIF) [file pone.0104392.s001.tif]
